# Sequential pancreatitis and diverticulitis following elective anterior lumbar disc replacement: a case report of a rare postoperative domino effect

**DOI:** 10.1097/RC9.0000000000000362

**Published:** 2026-03-09

**Authors:** Luthfi Gatam, Kemas A.M. Luthfi, Phedy Phedy, Karina Sylvana Gani, Mitchel Mitchel, Erica Kholinne

**Affiliations:** a Department of Orthopedic and Traumatology, Fatmawati Hospital, Jakarta, Indonesia; b Department of Orthopedic and Traumatology, Gatam Institute, Tangerang, Indonesia; c Department of Orthopaedics and Traumatology, Siti Fatimah Regional General Hospital of South Sumatra Province, Palembang, Indonesia; d Department of Surgery, Faculty of Medicine, Universitas Trisakti, Jakarta, Indonesia

**Keywords:** acute pancreatitis, case report, diverticulitis, lumbar disc replacement, peritonitis, postoperative complications

## Abstract

**Introduction:**

Anterior lumbar disc replacement (LDR) is a motion-preserving alternative to fusion for degenerative disc disease but carries risk of vascular and visceral injury. We report the first documented case of a sequential “domino effect” of acute postoperative pancreatitis followed by perforated diverticulitis after LDR surgery.

**Case presentation:**

A 66-year-old man with diabetes underwent elective L2–L3 LDR via a left anterior retroperitoneal approach. Although initially stable, he developed acute pancreatitis on postoperative day (POD) 1, confirmed by elevated enzymes and CT findings. Despite conservative treatment, recurrent fever and pain occurred, and by POD15, he developed peritonitis from perforated diverticulitis requiring open laparotomy and Hartmann colostomy. He recovered with multidisciplinary care.

**Clinical discussion:**

This case illustrates a possible “second-hit” mechanism where postoperative inflammatory stress triggered pancreatitis and subsequent colonic perforation.

**Conclusion:**

Postoperative abdominal pain following anterior lumbar surgery should not be dismissed as simple ileus. A high index of suspicion for rare but severe complications such as pancreatitis and diverticulitis, supported by early imaging, laboratory evaluation, and multidisciplinary team involvement, is essential. This case also advocates for selective preoperative abdominal CT in high-risk patients to aid surgical planning and mitigate catastrophic outcomes.

## Introduction

Lumbar disc replacement (LDR) is a motion-preserving alternative to fusion for degenerative disc disease (DDD), offering reduced adjacent segment degeneration^[^1,2^].^ However, the anterior retroperitoneal approach risks injury to great vessels and nearby organs. While vascular injury is a known but rare complication, pancreatic injury is exceptionally uncommon, with only few reported cases^[^3^]^.

Postoperative pancreatitis (POP) is exceedingly rare after spinal surgery, possibly due to ischemia, direct trauma, drug toxicity, or systemic inflammation^[^4^]^. Likewise, postoperative diverticulitis is scarcely reported, potentially triggered by altered motility, opioid use, or autonomic disruption^[^5^]^.

HIGHLIGHTS

- Acute pancreatitis can develop even after non-abdominal spinal surgery due to systemic inflammation.
- Early imaging, enzyme evaluation, and coordinated multidisciplinary care are vital to avoid severe postoperative complications.
- Postoperative inflammatory stress may trigger a “domino effect” of distant organ dysfunction.
- Pancreatitis-induced ileus and ischemia may lead to colonic diverticulitis and perforation.

We present a unique case of a sequence of acute pancreatitis (AP) followed by diverticulitis after an elective orthopedic procedure has never been reported. This case report details this unique “domino effect” in a patient after L2–L3 LDR, provides a comprehensive discussion of the potential pathophysiological mechanisms with a focus on anatomical and pathophysiological confirmation from the literature, and underscores the imperative for multidisciplinary management and potential preoperative screening. A contemporary review of the literature is also presented. The current study has been reported in accordance with SCARE 2025 standards^[^6^]^.

## Case presentation

A 66-year-old man presented to the orthopedic outpatient clinic with complaints of low back pain and bilateral anterior thigh pain worsened over several days, with symptoms exacerbated by prolonged standing and walking and partially relieved by rest. He denied recent trauma, fever, weight loss, bowel or bladder dysfunction, or constitutional symptoms. There was no history of acute abdominal pain or gastrointestinal complaints at presentation. He had previously been treated at a local clinic with anti-inflammatory medication, which provided minimal relief. His surgical history included multiple spinal procedures at other hospitals, namely a transforaminal lumbar interbody fusion performed in April 2023 and a spinal endoscopy in February 2025. The patient had no known drug allergies and denied alcohol use, smoking, or illicit drug use. Physical examination revealed bilateral hyperesthesia along the L3 dermatome, with preserved motor strength and a full range of motion.

**Figure 1.**
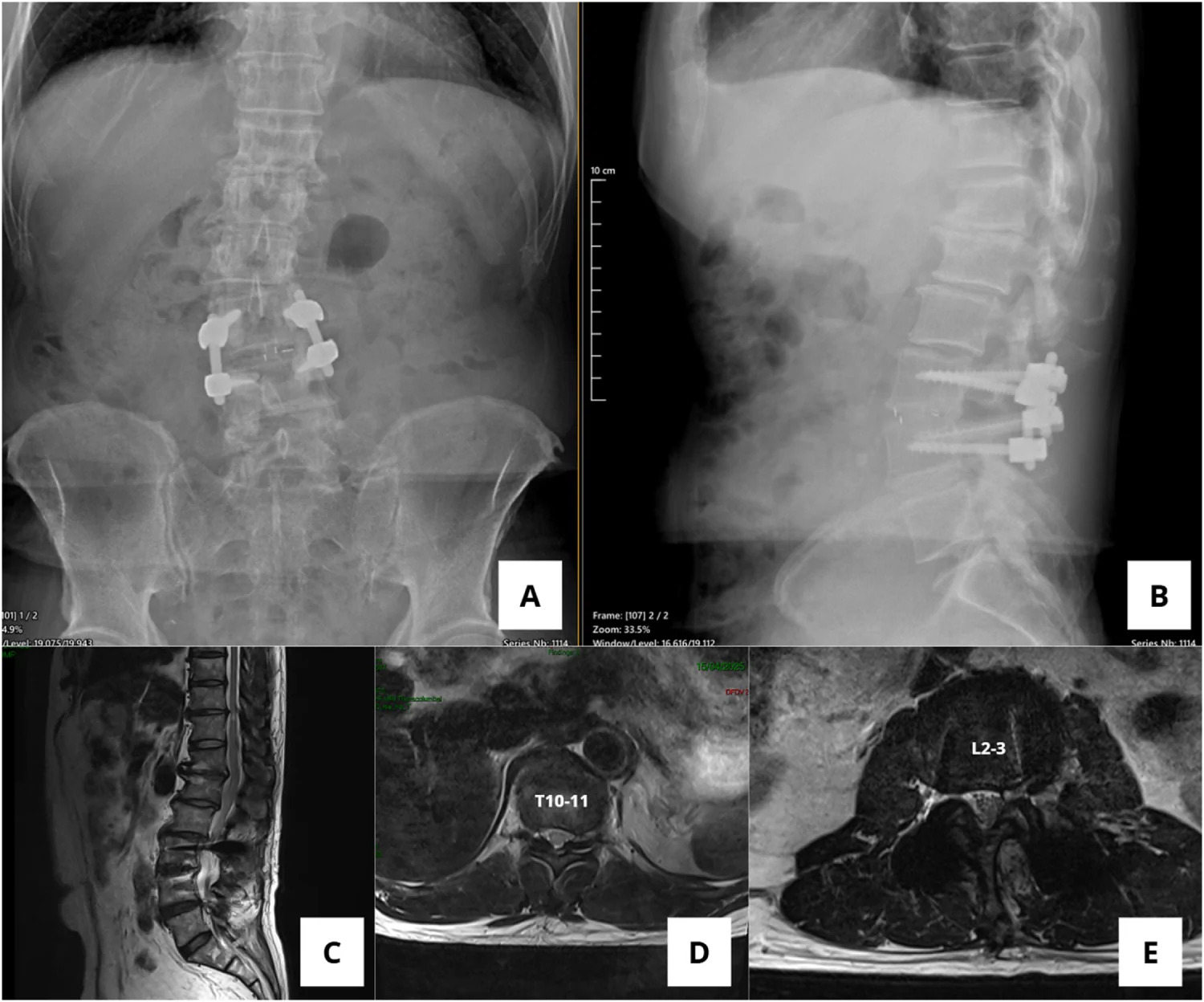
Preoperative imaging showing (A and B) X-ray AP and lateral view demonstrated pedicle screw and rod fixation at L3 and L4 with interbody fusion from a previous operation. Preoperative MRI T2-weighted images showing (C) spondylolisthesis at L2–L3 Meyerding Grade 1 and degenerative disc disease at L2–L3. (D) Axial view of T10–T11 showed spinal canal stenosis at T10–T11 Schizas grade B. (E) Axial view of L2–L3 showed spinal canal stenosis at T10–T11 Schizas grade B.

**Figure 2.**
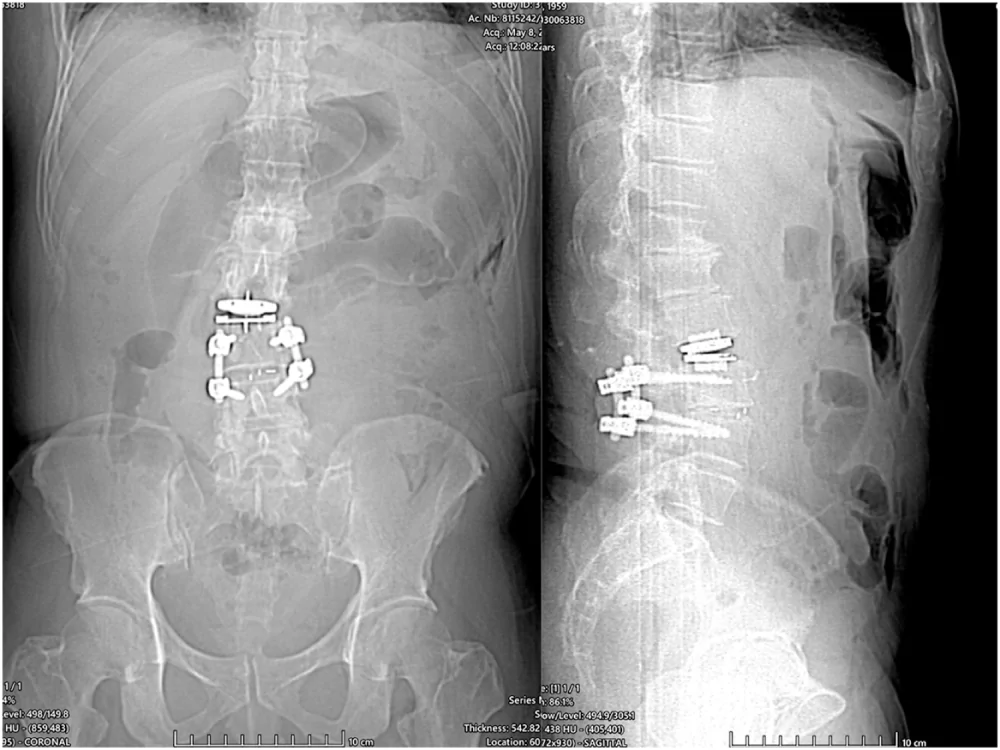
Postoperative X-ray showed L2–L3 lumbar disc replacement.

Imaging studies, including conventional radiographs and magnetic resonance imaging (MRI), demonstrated L2–L3 spondylolisthesis (Meyerding Grade 1), spinal canal stenosis at L2–L3 and T10–11 (Schizas Grade B), and DDD at L2–L3 (Fig. 1). We performed a LDR at L2–L3 (Fig. 2) and unilateral biportal endoscopy at T10–T11 under general anesthesia. The patient was positioned in the supine position under standard sterile operative fashion with a left pararectal approach. The LDR operative time was 103 minutes using a conventional anterior lumbar interbody fusion technique.

Intraoperatively, the patient required transfusion of 600 mL of autologous blood from a cell saver, three units of homologous blood, and 600 mL of fresh-frozen plasma due to blood loss. The procedure was otherwise uneventful, and the patient was transferred to the postoperative intensive care unit (ICU) for monitoring after awakening and maintaining spontaneous respiration.

## Postoperative course

**Figure 3.**
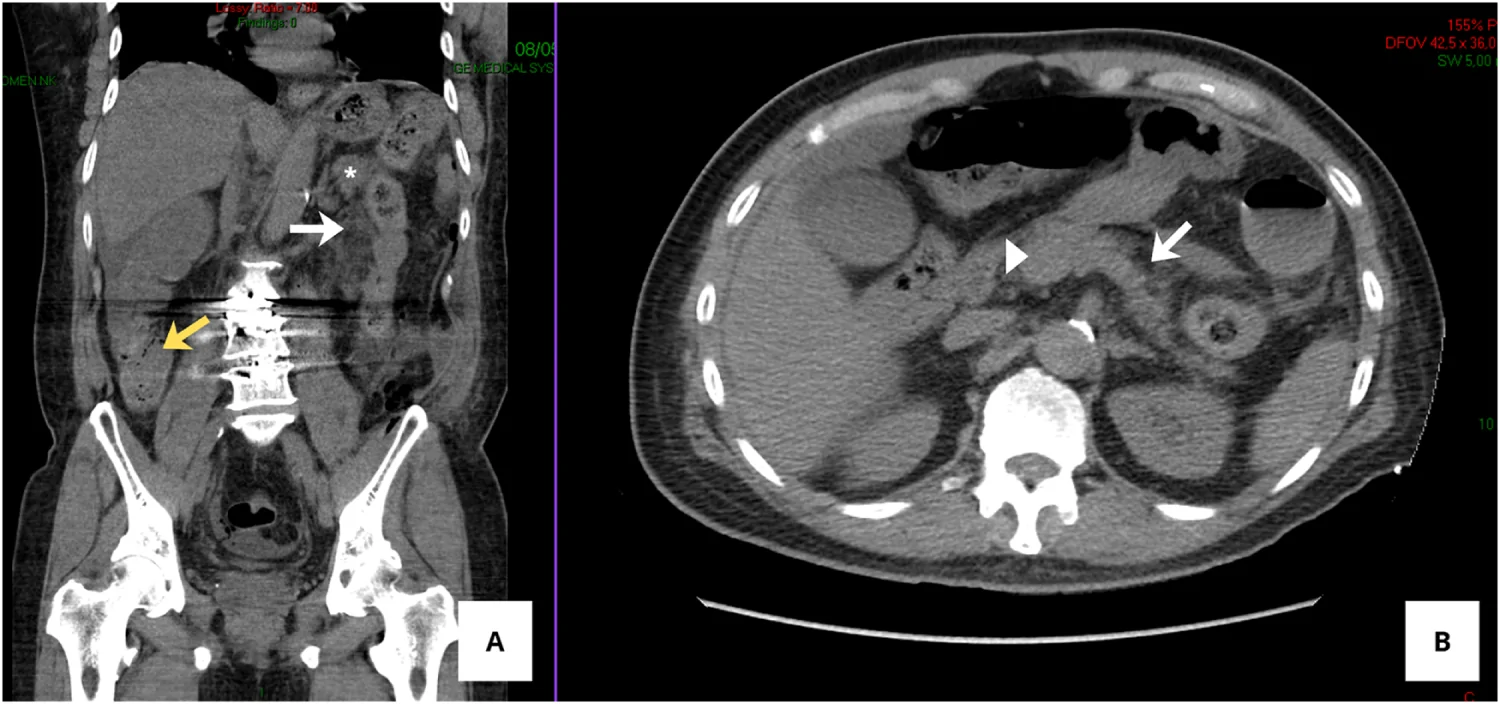
Non-contrast CT scan showing (A and B) an axial and coronal view of acute pancreatitis with pancreatic enlargement (white head arrow) and peripancreatic fat stranding (white arrow) around the body (white head arrow) and tail region (asterisk). There is also a small diverticulum at the ascending colon (yellow arrow) with no inflammatory changes.

**Figure 4.**
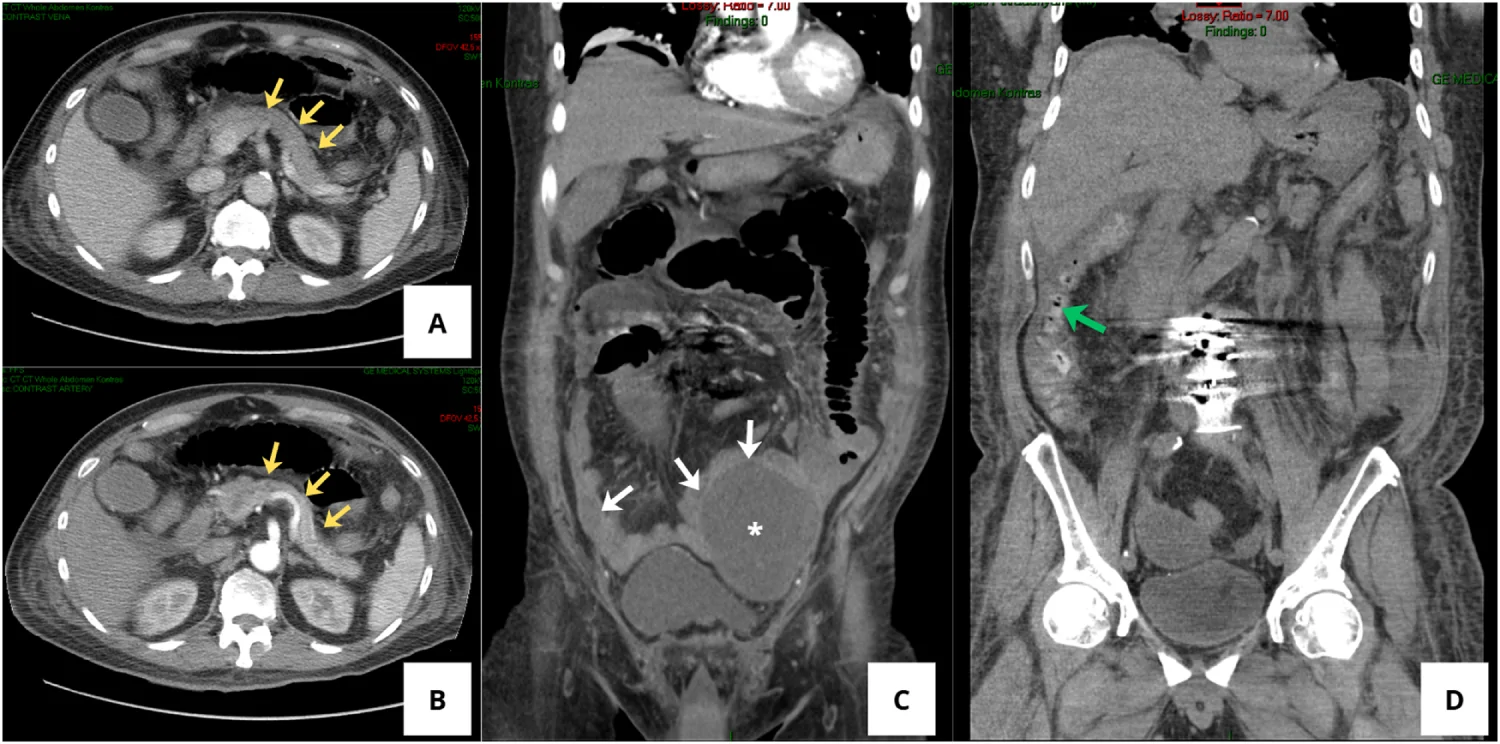
Intravenous contrast-enhanced CT scan of the abdomen showing (A and B) pancreatitis with fluid collection and peritoneal thickening (yellow arrow). (C and D) Non-contrast CT scan showing multiple walled-off pancreatic necrosis (asterisk and white arrow) in the pelvis and diverticulosis of the ascending colon (green arrow).

**Figure 5.**
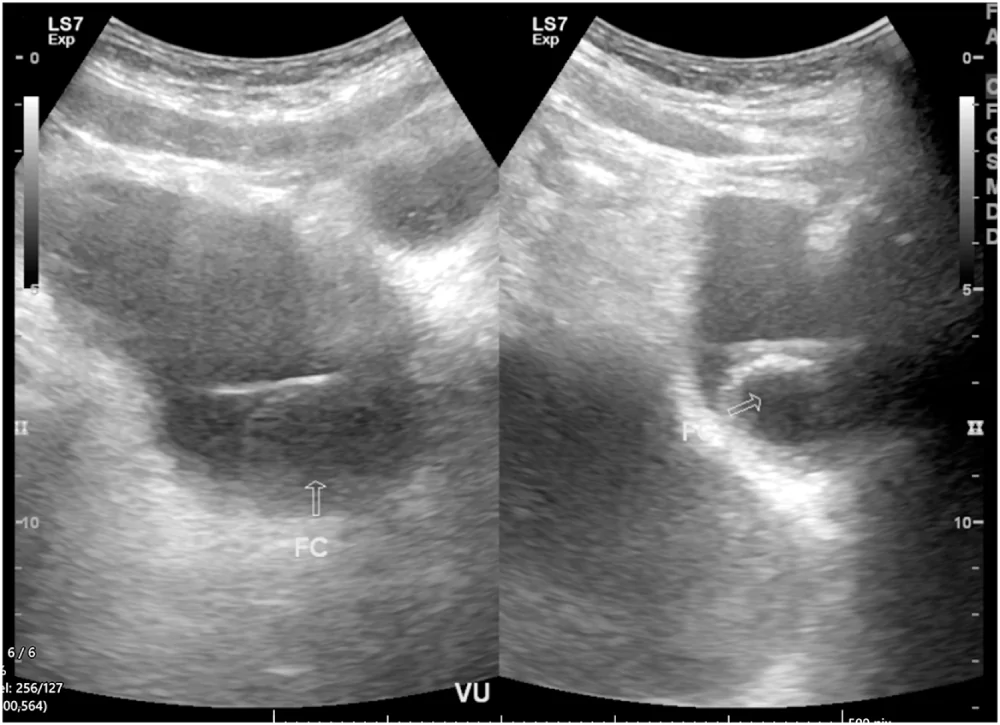
Abdominal ultrasound showed a well-defined hypoechoic fluid collection (FC) with internal debris consistent with a peritoneal abscess formation at the left suprapubic paramedian region.

In the immediate postoperative period, the patient was fully awake and exhibited no new neurological deficits. Pain was managed using a patient-controlled morphine infusion during the first postoperative day. On the first postoperative day, the patient experienced fever, severe abdominal pain, nausea, and vomiting. As these symptoms persisted into the second postoperative day, further laboratory evaluation was performed, revealing elevated reactive C-protein (214 mg/L), procalcitonin (7.57 ng/mL), lipase (195 U/L), and amylase (475 U/L) levels. A non-contrast abdominal CT scan confirmed the diagnosis of AP, showing pancreatic enlargement and peripancreatic fat stranding (Fig. 3). Differential diagnoses including postoperative ileus, intra-abdominal sepsis, and ischemic colitis were considered. These were excluded based on serial imaging, laboratory findings, and clinical progression, leading to the diagnoses of AP. The patient received aggressive fluid resuscitation along with somatostatin analog and broad-spectrum antibiotics. After several days of intensive care monitoring, the patient gradually improved over the next 8 days. However, on postoperative day 11, he developed recurrent abdominal pain and fever following the resumption of oral intake. A follow-up contrast-enhanced abdominal CT scan demonstrated AP with fluid accumulation, peritoneal thickening, and suspected multiple walled-off pancreatic necroses (WOPN) in the pelvic region, along with diverticulosis of the ascending colon (Fig. 4). Despite targeted antibiotic therapy with Tazocin, his clinical condition showed no significant improvement, and jaundice subsequently developed. Despite improvement in pancreatitis, the patient continued to experience fever and showed persistently elevated inflammatory markers. Two weeks after surgery, an ultrasound examination revealed complex ascites and a left suprapubic collection containing internal debris, suggestive of a developing peritoneal abscess (Fig. 5). These findings indicated progression to infected WOPN with reactive peritonitis, consistent with previous CT findings. Given the patient’s clinical decline and lack of response to conservative management, open laparotomy was undertaken the following day. Extensive debridement of necrotic and purulent tissue was carried out, followed by the creation of a diverting colostomy to achieve fecal diversion and control sepsis. Multiple drains were inserted, and the peritoneal cavity was thoroughly irrigated. Postoperatively, the patient was managed in the ICU. His condition gradually improved with supportive care and antibiotics, as drain output decreased, inflammatory markers normalized, and the colostomy became functional. A follow-up contrast-enhanced CT scan on postoperative day 18 demonstrated resolution of the pelvic abscesses and absence of pneumoperitoneum. The patient tolerated all interventions well during the hospital stay, with no evidence of non-adherence. Adherence was assessed through direct clinical observation and nursing documentation. The patient was subsequently discharged in stable condition and continued with outpatient rehabilitation. Follow-up evaluations at 1week and 1 month postoperatively revealed no complications.

## Discussion

Postoperative acute pancreatitis after spinal surgery is exceedingly rare, typically described only in isolated case reports^[^7^]^. Large series show the incidence to be well below 1%; for example, one study found a 0.10% occurrence of pancreatitis among 20 929 lumbar fusions^[^8^]^. When it does occur, the etiology is likely multifactorial. Proposed mechanisms include systemic inflammatory stress (surgical SIRS) and splanchnic hypoperfusion from intraoperative hypotension or blood loss^[^9^]^. Pancreatic ischemia may be exacerbated by patient factors (e.g., low body mass index) and direct pressure or traction on the pancreas during long spinal instrumentation^[^10,11^]^. In our patient, massive intraoperative hemorrhage requiring transfusion likely precipitated pancreatic injury. Type 2 diabetes itself is also a known risk factor for AP, roughly doubling AP risk in some studies, possibly due to microvascular dysfunction and metabolic effects. Elevated inflammatory markers (e.g., IL-6 and procalcitonin) in the early postoperative period support the notion that a “second-hit” SIRS response may have primed the pancreas for injury despite the surgery being remote from the abdomen^[^10,11^]^.

Importantly, this case illustrates a “domino effect”; AP can trigger profound gastrointestinal dysmotility; and enzyme-rich pancreatic inflammation may spread retroperitoneally, leading to paralytic ileus or even colonic obstruction^[^12,13^].^ The resulting ileus and distension promote bacterial overgrowth and increased intraluminal pressure^[^13–18^]^. In this milieu, pre-existing diverticula are vulnerable: current understanding holds that diverticulitis arises from fecal impaction and luminal obstruction within a diverticulum, leading to local stasis, ischemia, and microperforation^[^12,13,19^]^. We propose that pancreatitis-induced ileus and SIRS created a “perfect storm” for such a process. Inflammatory cytokines (TNF-α, IL-1β, and IL-6) likely impaired colonic microcirculation and cholinergic motility pathways, thereby compounding opioid analgesia and prolonging colonic stasis. This sequence, can rapidly escalate an asymptomatic diverticulum into an inflamed and eventually perforated diverticulitis^[^14–18^]^.

Pancreatitis induces a systemic inflammatory response and splanchnic hypomotility, disrupting normal gastrointestinal motility. This leads to paralytic ileus and gut stasis, which promote bacterial overgrowth and elevate intraluminal pressure^[^13,14^]^. In the presence of diverticula, these conditions can result in fecal impaction within the diverticular sac, causing localized ischemia and ultimately microperforation. This pathophysiologic cascade is supported by literature on diverticular disease^[^14–16^]^. Notably, diabetes and cardiovascular disease have been identified as risk factors for diverticulitis, suggesting that our patient’s diabetic microangiopathy may have further predisposed him to colonic ischemia under stress^[^15^]^. Although postoperative diverticulitis is scarcely reported (most series focus on immediate surgical complications), the chronology and imaging in our case strongly suggest that pancreatitis directly contributed to colonic compromise. No prior reports have documented this exact sequence of events after non-abdominal spinal surgery, underscoring its uniqueness.

This case underscores the importance of prompt, multidisciplinary management. Current guidelines for intra-abdominal sepsis and severe pancreatitis emphasize early diagnosis, aggressive source control, and coordinated care among surgical and critical care teams^[^20^]^. In our patient, early CT and laboratory evaluation enabled the diagnosis of pancreatitis on POD1, and surgical intervention with a Hartmann procedure provided definitive source control when peritonitis developed, consistent with contemporary recommendations^[^20^]^. Spine surgeons should not dismiss postoperative abdominal pain after anterior lumbar surgery as benign ileus. Even in the absence of direct abdominal injury, pancreatitis and secondary diverticulitis may occur through inflammatory or ischemic mechanisms. Early imaging, enzyme assessment and multidisciplinary sepsis management are essential to prevent life-threatening complications. To our knowledge, this case represents a previously unreported sequential occurrence of POP followed by perforated diverticulitis after non-abdominal spinal surgery, highlighting an under-recognized risk of serious abdominal complications in this setting. However, this report describes a single, rare clinical case and therefore cannot be generalized. A definitive causal relationship between POP and subsequent diverticular perforation could not be established; however, the temporal sequence, imaging findings, and clinical course suggest a biologically plausible association supported by existing literature.

## Conclusion

This case underscores the critical need for spine surgeons to maintain a high degree of clinical vigilance when assessing postoperative abdominal pain following anterior lumbar surgery. Even in the absence of direct abdominal intervention, rare but serious complications such as AP and diverticulitis can arise via systemic inflammatory and ischemic mechanisms. The observed “domino effect” illustrates how surgical stress may trigger a cascade of distant organ dysfunction. Timely diagnosis through early imaging and laboratory work-up, coupled with rapid, multidisciplinary intervention, is essential to preventing morbidity and optimizing patient outcomes in such complex postoperative scenarios.

## Data Availability

The data supporting the findings of this study are available from the corresponding author upon reasonable request.
